# Making Sense of Sexual Rights of the Disabled in Today’s Social–Cultural–Digital World

**DOI:** 10.1007/s10508-023-02585-8

**Published:** 2023-03-31

**Authors:** Carmen Yau

**Affiliations:** Faculty of Education, Health & Human Sciences, School of Human Sciences, The University of Greenwich, Old Royal Naval College, Park Row, London, SE10 9LS UK

Benoit et al.’s (2022) Target Article provides an intensive scoping review to explore “sexual assistance” as a contentious service option to actualize sex rights of people living with disabilities (PLWD). The Target Article identifies the breadth of the academic scholarship and categorizes them as a sex-positive cultural script and a sex-negative cultural script. Regarding the sex-negative cultural script, Benoit et al. highlight the impact of ableism and the medical model in shaping the negative assumptions and concerns aimed at safeguarding people living with disabilities. Regarding the sex-positive cultural script, Benoit et al. raised the assumption that PLWD have equal rights as the majority of people. In general, Benoit et al.’s Target Article is insightful and useful for readers looking to better understand discussions about the sexual rights of people living with disabilities. This Commentary aims to make sense of disability, sexual assistance, and sexual citizenship of PLWD in today’s social–cultural–digital era.

## Understanding Today’s Digital Era

In light of rapid development of digital technology and smartphone, social media is a new context for social interactions as people no longer require face-to-face engagement (Caron, 2016). Numerous scholars acknowledge that digital media and online communication welter us with different knowledge, cultures, religions, beliefs, values, and lifestyles. Individuals exercise their agency differently in the socio-cultural-technology context. With the emergence of smartphones, the modern world is digitally and technologically mediated (Lupton, 2015). Jurgenson (2012) coined the term “augmented co-reality” to describe how social media becomes an extension of everyday life. Gottschalk and Whitmer (2013, p. 328) state “since what happens online does not stay online, we must constantly resolve the tensions between the infinity of interactional opportunities our online life provides us, and the offline consequences of enjoying them.” Social media allows us to interact with people from different cultures and explore alternatives to experience and explore different social experiences.

## Making Sense of Disability and Disembodiment

In Benoit et al.’s (2022) Target Article, they highlight the elements of impairment and functionality in the definition of disability. The contextual factors of disability are briefly highlighted by citing the 2002 version of the International Classification of Functioning, Disability, and Impairment (ICF). Yet, the concept of disability should include the social and cultural factors. Disability is a “dynamic interaction between a person’s health condition, environmental factors and personal factors” (World Health Organization, 2013, p. 5). Besides an individual’s health conditions attributed to one’s lived experience of disability, the sociocultural context makes a significant impact on the experience and extent of disabilities. In today’s digital-saturated society, the world is intertwined with the physical environment and the virtual space. The inaccessible physical world hinders the full participation of PLWD, but the virtual world provides a safe and open space for effective social participation.

In today’s digital era, the disembedding force shakes the normative and traditional meaning of disability. As Giddens (1991) states, we are in the transition from a modern era to a post-modern era. Disability is socially constructed with stigmatization and prejudice. The negative stereotype of disability is normalized in the oppressive treatment of PLWD. PLWD themselves internalize these maltreatment and unequal social practice (Yau, 2019). Since the 1970s, the stigmatized ideology of disability began to shake as the disability rights movement and “crip” culture has grown. More writings and literature have made a significant change from a medical model that views disability as a pathological health condition to a social model that views disability as forms of diversities. The cultural symbol "disability" had a drastic change to form the concept of disability pride (aka “crip”). Disabled people positively unfold the meaning of disability with pride, similar to other minority groups like gay pride and black pride.

The concept of disembodiment implies people no longer make meanings from their bodily features in social experience. Some studies have begun to investigate how disabled people construct their identity on an online dating platform. Since the sexuality of disabled people is being stigmatized and delegitimized by dominant discourse in daily life, they tend to use social media to express their marginalized sexuality (Hall, 2018). Haraway (1994) found that disabled people minimize the limitation of their organic characteristics by re-constructing their self-identities and re-crafting their bodies in a virtual platform like *Second Life*. *Second Life* is prevalent in the Western disability community. Users are allowed to customize their avatar and engage in all sorts of social activities, including shopping, leisure, and sexual interactions. In a study on the psychological impact of *Second Life*, users with disabilities were motivated to engage as a world for better self-discovery, leisure, socialization, and equality. Users have a higher quality of life, better self-esteem, and other positive psychological outcomes (Kleban & Kaye, 2015).

On these dating platforms, PLWD decide how and when to disclose their disability during the relationship formation for courtship and intimacy (Saltes, 2013; Theodorou & Mavrou, 2017). PLWD strategically select, package, and control personal information disclosure in terms of their photos, descriptions, and background. Their self-presentation strategies are closely related to how they deal with existing stigmatization, social rejections, and a sense of self (Saltes, 2013). Saltes found that although the overall online dating experience of PLWD was a mixture of frustration and satisfaction, online dating was advantageous for PLWD to pursue desired social encounters. Benefits included extending broader social connections with others, privacy and anonymity, effective communication, and an open environment with more acceptance of disabilities (Saltes, 2013).

## Making Sense of Sexual Assistance

In Benoit et al.’s (2022) Target Article, they categorized articles into “sex-negative” vs. “sex-positive” perspectives to focus the discussion on sexual assistance. The “sex-negative” perspective features the medical model and justifies the social exclusion and safeguarding issues. Crawford and Ostrove (2003) attributed “infantilization” to disabled women who are socially constructed as incapable, dependent, socially isolated, and perceived as “asexual.” People tend to underestimate PLWD’s potential and capacity to take adult roles (e.g. sexual partners and mothers) in the future (Campbell, 2017). PLWD are marginalized, less respected, and less likely to receive consent in sexual relationships. Numerous studies reported that disabled women have a higher risk of sexual violence (Barrett et al., 2009; Brown et al., 2017; Brownridge, 2006; Hasan et al., 2014).

Social and health services shape the perception of disabled bodies and form how disabled bodies should be treated in professional practice. Physical appearance and body have rigid social norms and expectations. These normative attitudes push women to fit in the dominant norm as “normal,” such as dressing up, putting on makeup, dieting or even surgical procedures. In an anthropology book *Venus on Wheels*, Frank (2000) reported how Diane DeVries, a woman born without arms and legs, was being constructed and treated negatively in hospital settings. It is a common practice among women with limb deficiencies that they are required to undergo material restoration through prostheses. As Frank commented, these medical practices reinforce the discrimination of disabled female bodies as “damaged” ones. This practice becomes an unpleasant and shameful experience among disabled women. This experience makes them loathe their body, their disability, and themselves.

The “sex-positive” perspective employs a social model to highlight social barriers and emphasizes the equal rights of PLWD. Social environments are another source of discourse representation on disability, closely connected to psychology (Kitchin et al., 1997). Inaccessibility and how the built environment is designed by/for non-disabled individuals dictate how disabled people are being presented in an unfriendly manner. Disabled women report that inaccessible and disability-unfriendly social infrastructure (e.g., transportation, accessibility of shopping malls, and places for social engagement) creates a sense of bias that people with disability are dependent on others and not engaging in adult social interactions (Sonali, 2017).

To conclude, in Benoit et al.’s (2022)’s Target Article, “sex-negative” dictates the sexuality of PLWD as inferior while “sex-positive” indicate the sexuality of PLWD as equal. I would have appreciated it more if Benoit et al. could have also reviewed some articles through the lens of crip culture. Using the lens of crip culture, the sexuality of PLWD is framed and projected as a sexual advantage as PLWD do not have to comply with the heteronormative gender and sexuality.

In crip theory, social deconstruction aims to question discourse on disability to break the oppression and leverage the privileges of living with a disability. Crip theory makes a significant impact on the discourse of sexuality of disabled people. Instead of subverting disability stigma, more disabled people are packaging their disability as a sex advantage (Guldin, 2000; Kaufman et al, 2007; O'Toole, 2000; Shakespeare et al., 1996). A remarkable example is the promotion of the sex advantage of disabled people. Connie Panzarino, a disabled lesbian, joined a pride parade wearing a sign that stated, "Trached^1^ dykes^2^ eat pussy all night without coming up for air" (O'Toole, 2000, p. 212).

## Making Sense of Sexual Citizenship

In Benoit et al.’s (2022) Target Article, sexual assistance is PLWD’s agenda to achieve sexual citizenship. Benoit et al.’s inclusion and exclusion criteria on “sexual assistance” embed a sense of ableism and heteronormative bias to include the diverse and comprehensive understanding of “sexual assistance.” Articles under review focus on PLWD as the recipient of a sexual service provider or personal assistant. Articles were excluded if the service provider was a PLWD or if other forms of sexual facilitation (e.g., self-stimulation) were used. In a similar vein, Kim (2010) criticized that sex trades and sexual assistance are usually framed as a sexual relief for PWLD and embed the assumption of heteronormativity and ableism. Furthermore, patriarchy implies the assumption that heterosexuality is normative, prescribed, and privileged. People perceive themselves and others through the lens of dominant presumptions of binary gender identity and heterosexual orientation. Heteronormative culture limits sex to goal-oriented penetration for reproduction and ignore non-heteronormative sexual pleasure.

The meaning of “sexual assistance” does not fully imply a give-and-take relationship. For instance, sex volunteers from Hand Angels in Taiwan highlight the mutual benefits and the political element (Yau, 2023). Sex volunteer service from Hand Angels does not aim to provide service as compensation for the structural oppression against PLWD nor for the realization of equal rights of PLWD. The goal of sex volunteers from Hand Angels is a social work practice across micro, mezzo, and macro levels. Their volunteer team is a mixture of PLWD and able-bodied members to provide comprehensive service from screening, needs assessment, service delivery, and critical reflection (Yau, 2023). The meaning of “sexual assistance” can be an experiential learning process for both PLWD and sex volunteers to learn and exercise their agency in engaging sexual and intimate relationships. Benoit et al.’s (2022) criteria reflect the biased assumption and oversimplification of the implementation and realization of sexuality of PLWD. Benoit et al. should have reviewed articles that related to service providers living with disabilities and alternative sexual facilitation. If Benoit et al. had done so, their Target Article could have further acknowledged the sexual agency of PWLD and the sub-culture of the digital world.

Benoit et al. (2022) focused on “sexual assistance” as part of the sexual citizenship of PLWD. Sexuality is the core of self-acceptance and acceptance by others as a mature and independent individual worthy of affection, intimacy, and love. Sexual identity is one of the extraordinary challenges for individuals “to express, to explore and to have positive validated” throughout their lives (McKenna et al., 2001, p. 302).

The term “sexual citizenship” involves a spectrum of participation and involvement from sex education, sex healthcare, and legal infrastructure. For example, the absence and inaccessibility of women’s services (e.g., gynecology and family planning services) hamper the sexual citizenship of disabled women. Inaccessibility further cultivates the normative attitude to exclude and devalue the rights of disabled women (Crooks & Chouinard, 2006). Sexual citizenship should include access to healthcare and include universal design of the clinic environment and facilities, and financial and informational resources (Anderson & Kitchin, 2000).

Thus, authenticity should be a crucial element of sexual citizenship among PWLDs. As Gerschick (2000, p. 1264) writes, “bodies are central to achieving recognition as appropriately gendered beings. Bodies operate socially as canvases on which gender is displayed and kinaesthetically as the mechanisms by which it is physically enacted.” Another good example is the new concept of "abstract orgasm." As Guldin (2000) reports, some disabled people experience erotic sensations and orgasm from alternative parts of their bodies and minds. Siebers (2012, p. 47) states that the sexuality of disabled people is currently creating "different conceptions of the erotic body, new sexual temporalities, and a variety of gender and sexed identities."

Guldin (2000) pointed out that disabled people continuously rebuff and unfold the dominant norm of sexuality by examining the idea of the esthetic body and the denotation of orgasms. Guldin shared that a female disabled participant identified herself as a slut. Guldin interpreted her “slut” self-identification as “enabling her to challenge the cultural de-sexualization of her body as well as that of her parents who told her that someday a man would love her enough to sleep with her despite the disability” (p. 237). A similar idea is supported by Siebers (2012, p. 47), who noted that disabled people hold “different conceptions of the erotic body, new sexual temporalities, and a variety of gender and sexed identities.” Taken together, disabled people face various challenges and barriers, but they are still able to express their sexuality in different forms and live healthy, adventurous, and satisfying sexual lives.

To conclude this commentary, Benoit et al.’s (2022) Target Article provides a comprehensive review of sexual assistance for PLWD. It helps to explore and give insights to discuss the advocacy of the sexual rights of PLWD. Gergen (1991, 2000) points out that technology enables individuals to relate to people from all paths of life. Technology and digital media become the catalyst for transformation of the meaning of community and individuality. It is necessary to include digitally mediated social encounters and interactions in our discussion of sexuality as individuals live in the digital era. Further studies should focus on the agency of PLWD to reach their sexual citizenship in today’s digital saturated context.

## Footnotes

1 Trached implies people who are mechanically ventilated and received a tracheostomy, a medical procedure to create an opening on the neck to insert a breathing tube into one’s windpipe.

2 Dykes is a slang implying lesbian.
